# Capture and *On-chip* analysis of Melanoma Cells Using Tunable Surface Shear forces

**DOI:** 10.1038/srep19709

**Published:** 2016-01-27

**Authors:** Simon Chang-Hao Tsao, Ramanathan Vaidyanathan, Shuvashis Dey, Laura G. Carrascosa, Christopher Christophi, Jonathan Cebon, Muhammad J. A. Shiddiky, Andreas Behren, Matt Trau

**Affiliations:** 1 Centre for Personalised NanoMedicine, Australian Institute for Bioengineering and Nanotechnology, University of Queensland; 2 School of Chemistry and Molecular Biosciences, The University of Queensland, Brisbane, Queensland, 4072, Australia; 3 Olivia Newton-John Cancer Research Institute, Heidelberg, Victoria, 3084, Australia; 4 Department of Surgery – Austin Health, University of Melbourne, Heidelberg, Victoria, 3084, Australia; 5 School of Cancer Medicine- La Trobe University, Melbourne, Victoria, 3086, Australia; 6 Ludwig Institute for Cancer Research – Austin Health, Heidelberg, Victoria, 3084, Australia

## Abstract

With new systemic therapies becoming available for metastatic melanoma such as *BRAF* and PD-1 inhibitors, there is an increasing demand for methods to assist with treatment selection and response monitoring. Quantification and characterisation of circulating melanoma cells (CMCs) has been regarded as an excellent non-invasive candidate but a sensitive and efficient tool to do these is lacking. Herein we demonstrate a microfluidic approach for melanoma cell capture and subsequent *on-chip* evaluation of *BRAF* mutation status. Our approach utilizes a recently discovered alternating current electrohydrodynamic (AC-EHD)-induced surface shear forces, referred to as *nanoshearing*. A key feature of *nanoshearing* is the ability to agitate fluid to encourage contact with surface-bound antibody for the cell capture whilst removing nonspecific cells from the surface. By adjusting the AC-EHD force to match the binding affinity of antibodies against the melanoma-associated chondroitin sulphate proteoglycan (MCSP), a commonly expressed melanoma antigen, this platform achieved an average recovery of 84.7% from biological samples. Subsequent staining with anti-*BRAF*^V600E^ specific antibody enabled *on-chip* evaluation of *BRAF*^V600E^ mutation status in melanoma cells. We believe that the ability of *nanoshearing*-based capture to enumerate melanoma cells and subsequent *on-chip* characterisation has the potential as a rapid screening tool while making treatment decisions.

Melanoma is the 4^th^ most common cancer in Australia and until recently, was commonly fatal after metastasizing beyond regional lymph nodes. Advances in the field have enabled the development of effective therapies, such as inhibitors that target oncogenic *BRAF* protein, the product of V600 mutations of *BRAF*. Patients with activating *BRAF* mutations constitute up to 50% of melanoma patients^1^,^2^ and frequently respond to BRAF-inhibitor treatment^3^. Unfortunately tumour responses to BRAF inhibitors only last around 6–9 month after which relapse commonly occurs^4^,^5^. Combination strategies such as a BRAF inhibitor plus a MEK inhibitor modestly extend the duration of tumour response^6^,^7^,^8^.

Currently the identification of patients with such mutations requires tumour biopsy and subsequent DNA analysis by sequencing or PCR amplification methodologies^9^. Biopsy material may not be readily available or accessible. Furthermore, when patients who have been receiving kinase inhibitors develop resistance to the treatment, assessment by biopsy to evaluate resistance can be invasive, time consuming and impractical^10^. Consequently the use of a reliable blood test to enable rapid analysis of *BRAF* mutation status and disease monitoring would be extremely valuable and has the potential to transform the current management of melanoma^11^.

Circulating melanoma cells (CMCs) have been suggested as ideal biomarkers for monitoring disease progression since their presence in the bloodstream is a pre-requisite for metastasis and their levels reflect response to therapy^12^,^13^. Furthermore, accessing CMCs provides a non-invasive means of characterising the tumour, and can reveal genotypic and phenotypic evolution during tumour progression, thereby assisting with the identification of potential new targets^14^,^15^. However, isolation and characterization of melanoma cells from complex biological samples present significant challenges since: (*i*) Melanoma cells in the circulation are present at very low frequencies (averaging 1–100 cells/ml) in comparison to peripheral blood cells (10^7^)^16^, and (*ii*) with epithelial cell adhesion molecule (EpCAM) forming the basis of most circulating tumour cells (CTCs) isolation strategies^17^,^18^, its absence on melanoma cells and other non-epithelial cancers highlights the need for additional markers and versatile platforms that can easily incorporate different markers^19^.

In recent years, several melanoma-specific cell surface molecules have been suggested for melanoma cell enrichment, including melanoma chondroitin sulphate proteoglycan (MCSP), melanoma cell adhesion molecule (MCAM) or CD271^20^. Among these, MCSP—also known as the high molecular weight-melanoma-associated antigen (HMW-MAA)—is expressed in majority (>85%) of melanoma cell types with limited intra- and inter-lesional heterogeneity. It also represents a potential candidate for immunotherapy targets^20^,^21^,^22^,^23^,^24^. Although it has been possible to isolate and subsequently analyse melanoma cells^25^,^26^, most methods require cells to be released in order to perform downstream analysis using conventional methodologies (i.e., DNA sequencing or RT-qPCR). Thus, an effective methodology that can enable simultaneous cell capture and direct cellular analysis would improve diagnostic effectiveness by reducing analysis time and assay complexity.

Herein, we report a simple microfluidic approach to sensitively enumerate melanoma cells by utilizing alternating current electrohydrodynamic (AC-EHD)-induced surface shear force, referred to as *nanoshearing*. The *nanoshearing* approach involves generation of shear forces acting within nanometers of the electrode surface to promote specific cell-antibody interactions whilst simultaneously displacing the weak non-specifically bound cells. This is achieved by adjusting AC-EHD force to select the magnitude of shear forces that maximizes specific binding capability of antibody-antigen interaction. In this study, we adjusted AC-EHD forces to enable effective capture of MCSP(+) melanoma cells, whose expression and genetic profiles have been well characterized^27^. This approach has proven to be effective in isolating high purity breast cancer cells as well as other biomolecular entities^28^,^29^.

Captured CMCs onto the *nanoshearing* platform were subsequently analysed *on-chip* for the presence of *BRAF*^*V600E*^ mutation using the anti-*BRAF* V600E specific antibody (VE1 clone)^30^. This antibody has previously been used for the reliable identification of this mutation in tissue samples, enabling us to circumvent the need for DNA sequencing^31^. However, for the first time, this antibody has been utilized in a microfluidic system to facilitate rapid mutation analysis.

## Results

### Determining the optimal AC-EHD operational parameters

**Figure 1:**
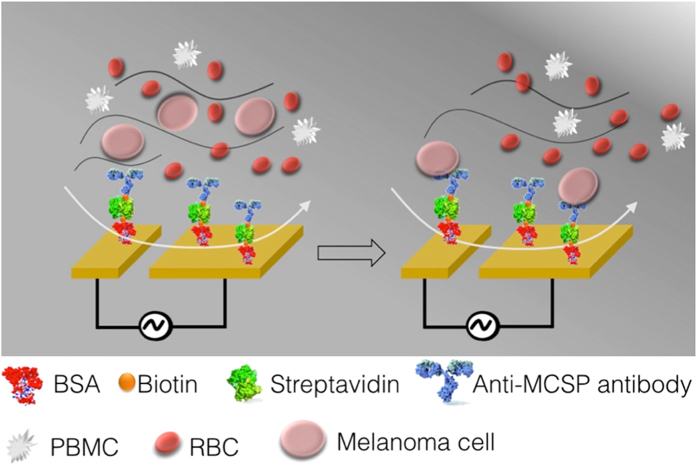
Mechanism of AC-EHD induced fluid flow for cell capture. The application of an AC EHD field, results in lateral fluid flow in the direction of the broken asymmetry (e.g., towards larger electrode). When samples containing target melanoma cells are driven through antibody-functionalized devices under AC-EHD flow, it provides the capability to specifically capture target cells by increasing the number of cell-antibody (surface bound) collisions, which is a result of improved analyte transport. Since the magnitude of this force can be *tuned* externally via the application of ac field, it can be applied to preferentially select specifically bound cells over nonspecifically adsorbed non-target species. (BSA- bovine serum albumin. RBC- red blood cell. PBMC- peripheral blood mononuclear cell.)

The use of electrically driven fluid flow represents a promising approach to induce fluid movement across microfluidic channels. Brown *et al*. and others have extensively utilized AC-induced fluid flow phenomena including AC electro-osmosis and dielectrophoresis for the manipulation of colloidal particles, nucleic acids as well as a wide range of cellular species on electrode surfaces^32^,^33^,^34^,^35^,^36^,^37^,^38^. In this study, we have adopted a simple microfluidic device utilizing AC-EHD induced fluid flow for the specific capture and enumeration of melanoma cells. To this end, we constructed a microfluidic device (Supplementary Fig. S1 for device design) containing a large array of asymmetric gold electrode pairs within a microfluidic channel. Figure 1 demonstrates AC-EHD induced fluid flow movement for melanoma cell capture. The large and small electrodes in each asymmetric electrode pair are considered to be cathode and anode of an electrolytic cell. Upon application of an AC field (*i.e.*, applied potential) across these electrode pairs, charges are induced within the electrical double layer on the electrode surface (double layer thickness = 2 nm for 1 mM PBS; calculated using Debye-Hückel approximation). Due to the asymmetric geometry, the lateral variation in total number of charges and their non-homogeneous spatial distribution within the double layer, gives rise to two non-uniform local forces with the force on the larger electrode being greater than the force on the smaller electrode (F_L_ > F_s_)^28^,^39^. The resultant force drives the fluid through the channel, across the antibody-functionalized electrode surface and facilitates enhanced cell-antibody interaction. These forces exist within nanometer distances from the electrode surface where cells come into contact with antibodies, therefore could also be harnessed to disrupt the weaker non-specific bonds and displace non-target cells.

The flow pattern under AC-EHD field is different from that of a laminar flow that has a parabolic flow profile within the flow channel (consistent with the “Poiseuille Law”^40^). This type of flow has a stationary boundary layer of fluid at the solid-liquid intereface. *Nanoshearing* is therefore an entirely different (electrohydrodynamic) effect, which causes forced motion of fluid within this traditionally stationary layer. The *nanoshearing* phenomenon causes the flow of fluid within a “Debye Length” distance from the surface of the electrode (between 1–5 nm for our systems) and is consequently entirely different to that of laminar flow. Our previous investigations on capture efficiency under different AC-EHD conditions in comparison with similar flow rates under hydrodynamic flow (*via* a syringe pump) demonstrate a significant enhancement in capture efficiency across all operating AC-EHD flow rates was observed in comparison to pressure driven flows^39^. This enhanced capture efficiency under AC-EHD induced fluid flow is presumably owing to the additional effective manipulation of shear forces (*i.e.*, *nanoshearing*) and concomitant fluid mixing that can augment the specific capture of cells due to increased number of effective cell-surface (antibody functionalized) collisions.

**Figure 2:**
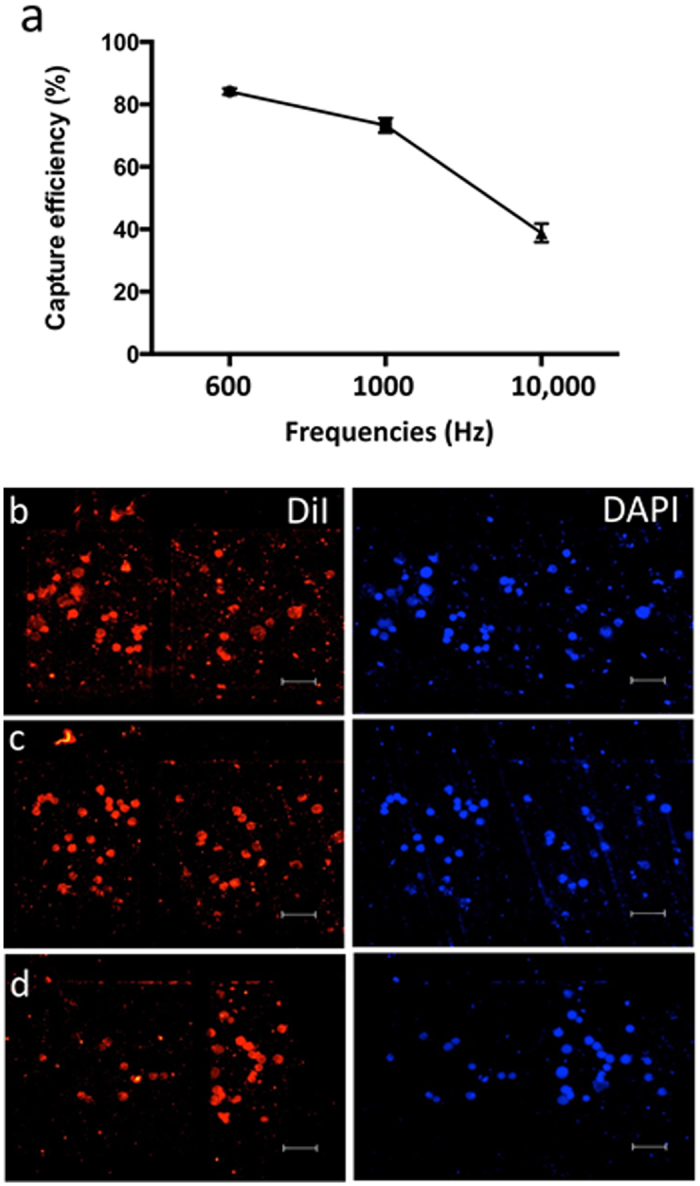
Effect of applied AC field on cell captures. (**a**) Capture efficiency from PBS (10 mM, pH 7.4) spiked with LM-MEL-6 (500 cells in 500 μL PBS) under the frequency range *f* = 600 Hz − 100 kHz at constant amplitude of *V*_pp_ = 100 mV. Each data point represents the average of three separate trials (*n* = *3*) and error bars represent standard error of measurements within each experiment. Representative fluorescence images of pre-stained (DiI-red) LM-MEL-6 cells spiked in PBS, and nuclear stain DAPI (blue) under the frequency of (**b**) *f* = 600 Hz, (**c**) 1000, and (**d**) 10000 kHz at *V*_pp_ = 100 mV. 10× magnification. Scale bar is 50 μm.

The critical parameters that influence fluid flow and hence the shear force is primarily determined by the AC-EHD force, which is the result of AC frequency and amplitude. To investigate the effect of different AC frequencies and hence the effect of resultant AC-EHD induced surface shear force on CMC capture, we examined the changes in capture efficiencies with alteration of AC frequencies using anti-MCSP functionalized devices (Supplementary Fig. S2). LM-MEL-6 cells were spiked in PBS, a media of considerably low conductivity than the culture media. As shown in Fig. 2a, the capture efficiency decreases as we increase the AC frequencies from 600 Hz to 10 kHz, indicating the capture performance of our devices is a function of applied AC-EHD force. Figure 2b–d shows representative fluorescence images of melanoma cells captured on the electrode under difference frequencies (b) *f* = 600 Hz, (c) 1000, and (d) 10000 kHz. The maximum capture efficiency of 84.1% was achieved under the applied field of *f* = 600 Hz and *V*_pp_ = 100 mV. This data was reproducible with a relative standard deviation (RSD) of 1.0% (*n* = *3*). Under this field conditions, capture efficiency is maximized probably due to the stimulation of the fluid flow around the anti-MCSP-functionalized electrodes, which can increase the effective antibody-antigen interactions (a condition where shear force < antibody-antigen binding force). In contrast, higher frequencies resulted in stronger fluid flow (a condition where shear forces > antibody-antigen interaction), which significantly reduced the antibody-antigen interactions. This capture trend was in line with our previous observations on the use of AC-EHD induced fluid flow for breast cancer cell capture and suggests that the cell-antibody binding can be influenced by manipulation of the shear forces within the double layer of the antibody-functionalized electrode surface^39^. In our previous study, the optimal frequency for capturing breast cancer cells (SK-BR-3) was determined to be 1000 Hz^39^. This higher value suggests that breast cancer cell to anti-HER2 antibody interaction had a larger binding force than melanoma cells to this particular MCSP antibody. It also demonstrates the flexibility of our platform to accommodate different cell types and antibodies by only tuning the AC-EHD field.

### CMCs isolation specificity

To determine the specificity of MCSP immunocapture, we conducted experiments on MCSP functionalized devices using two separate cancer cell lines that did not express MCSP; the melanoma cell line LM-MEL-75 and the breast cancer cell line SK-BR-3. A total of 1000 cellsmL^−1^ of LM-MEL-75 and/or SK-BR-3 cells were spiked in PBS and run on *nanoshearing* devices under an applied AC-EHD force of *f* = 600 Hz and *V*_pp_ = 100 mV. Negligible levels of nonspecifically adsorbed cells (1.4 ± 0.2% of MCSP(−) and 0.9 ± 0.2% of SK-BR-3 cells) were observed for both cell lines (Supplementary Fig. S3). Similarly, analysis of MCSP(+) melanoma cells (LM-MEL-6) at 2000 cellsmL^−1^ concentration using devices functionalized without anti-MCSP antibody under the same AC-EHD force, also resulted in a negligible level of nonspecifically adsorbed cells (0.23 ± 0.09%). This level of background response from nonspecific cells indicates that our device was highly specific at recognizing target MCSP cells.

### Analytical performance of the device

**Figure 3:**
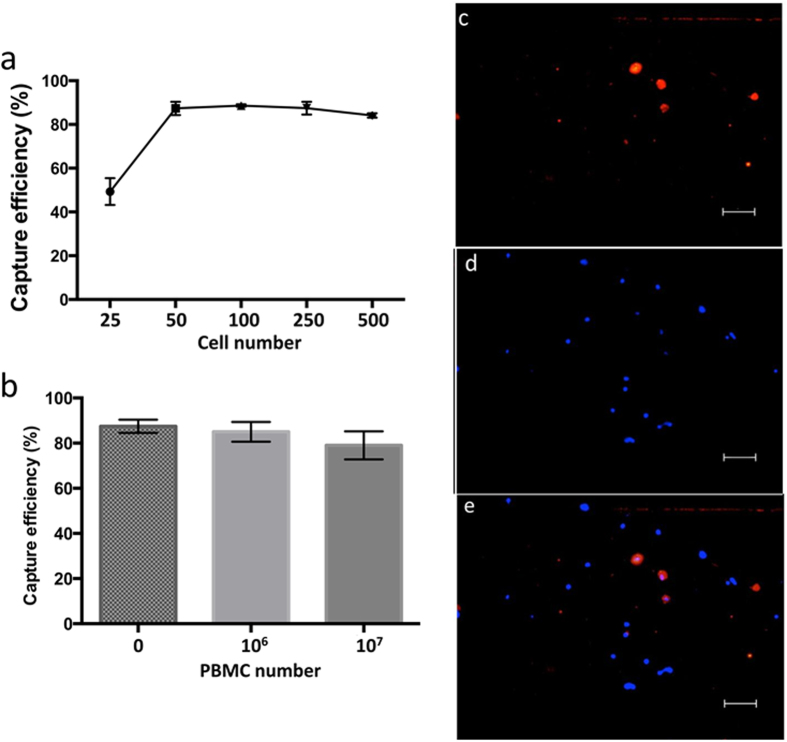
Capture performance of AC-EHD device. (**a**) Capture efficiency from PBS spiked with pre-stained 25–500 LM-MEL-6 cellsmL^−1^ under AC-EHD flow conditions. (**b**) Capture efficiency from PBS spiked with pre-stained LM-MEL-6 (250 cellsmL^−1^) along with 0, 10^6^ and 10^7^ PBMCs under AC-EHD flow conditions. Each data point in (**a**,**b**) represent the average of three separate trials (*n* = *3*) and error bars represent standard error of measurements within each experiment. (**c**–**e**) Representative fluorescence images of pre-stained LM-MEL-6 cells (100 cellsmL^−1^) spiked in PBS along with 10^6^ PBMCs - (**c**) DiI-red, (**d**) nuclear stain DAPI (blue) and (**e**) DiI+ DAPI. 10× magnification. Scale bar is 50 μm. Data presented in (**a**–**e**) were obtained using the AC-EHD force of *f* = 600 Hz and *V*_pp_ = 100 mV.

In order to determine the dynamic range of detection of our device, PBS samples containing spiked MCSP(+) LM-MEL-6 cells ranging from 25 to 500 cells were driven through anti-MCSP functionalized devices (Fig. 3a and Supplementary Fig. S4). Under optimal AC-EHD conditions (*f* = 600 Hz and *V*_pp_ = 100 mV), the average recovery for the seed level of 50–500 cellsmL^−1^ was 86 ± 1.48%. However, reduced capture efficiency was found for a seed level of 25 cellsmL^−1^ (49.3 ± 3.52%). These data suggest that our device is capable of capturing at least 50 cellsmL^−1^ with >86% capture efficiency.

To investigate the fidelity of our method in detecting melanoma cells with different MCSP expression level, cells from a low MCSP expressing cell line (LM-MEL-53) were used (Supplementary Fig. S5a for FACS analysis). Designated concentrations (100 and 50 cellsmL^−1^) of LM-MEL-53 and/or LM-MEL-6 were spiked in PBS and processed through anti-MCSP functionalized device under optimal AC-EHD flow (*f* = 600 Hz and *V*_pp_ = 100 mV). As can be seen from Supplementary Fig. S5b, the device performance was consistent for both cells lines at each of the tested concentrations. These observations were also supported by statistical analysis (p = 0.14 (LM-MEL-53) and p = 0.23 (LM-MEL-6), for 100 and 50 cellsmL^−1^, respectively) across these concentrations. The average capture efficiencies for LM-MEL-6 were 88.7 ± 0.6% and 87.3 ± 3.1% for 100 and 50 cellsmL^−1^ respectively. Similarly, the average capture efficiencies for LM-MEL-53 were 85.3 ± 3.1% and 84 ± 2.6% for 100 and 50 cells respectively. This indicates that our device performance was unperturbed by the difference in expression levels of MCSP in melanoma cells. This can be attributed to the synergistic effect of the capture agent (*e.g.*, anti-MCSP antibody) and the ability of surface shear forces to enhance the frequency of antibody-cell collisions.

To investigate the potential of this approach for capturing CMCs from biological fluids, blood samples were simulated by spiking 100 MCSP(+) LM-MEL-6 cells into 1000μL of PBS containing 10^6^ or 10^7^ peripheral blood mononuclear cells (PBMCs) isolated from healthy donor blood. Samples were run through the functionalized device under optimal AC-EHD field. The capture efficiency remained similar (84 ± 2.6%) in case of samples containing 10^6^ PBMCs whilst a slight decrease in cell recovery (79 ± 6.2%) was observed with an increase in the number of PBMCs to 10^7^ (Fig. 3b). Furthermore, the average purity of the isolated cell population (% purity = % captured tumour cells /% non-specific cells) was significantly large, in the range of 20% (22.4 ± 3.0% (10^6^ PBMCs) and 19.1 ± 1.6% (10^7^ PBMCs)). Figure 3c,d shows representative fluorescence images of melanoma cells and PBMCs that were captured on the electrode.

### *On-chip* immunofluorescence detection of *BRAF* ^V600E^ mutation

**Figure 4:**
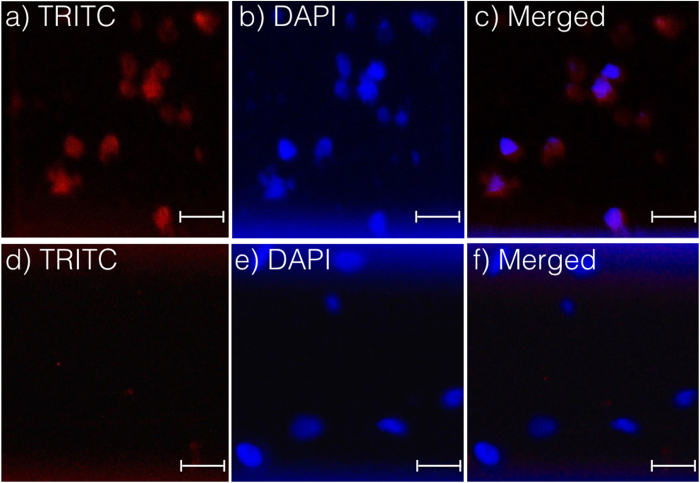
*BRAF*^V600E^ antibody staining. Images (**a**–**c**) show LM-MEL-6 (BRAF^V600E^ positive) cells stained with (**a**) anti-*BRAF* V600E antibody (with Alexa Fluor 555 secondary antibody) and (**b**) DAPI after been captured on the *nanoshearing* device. Images (**d**,**e**) show LM-MEL-53 (BRAF^V600E^ negative) cells stained with the same antibodies but did not show any Alexa Fluor 555 signals from cells illustrating absence of V600E. 20× magnification. Scale bar is 50 μm.

To demonstrate *on-chip* detection of *BRAF* mutations across the melanoma cell population, we spiked 100 LM-MEL-6 (*BRAF*^V600E^) and LM-MEL-53 cells (*BRAF* wild type) in PBS, and captured them under optimal AC-EHD force. Following membrane permeabilisation and nucleus staining of captured cells using DAPI, their mutational status was established by staining with anti-*BRAF*^V600E^ primary antibodies and fluorescent labelled secondary antibodies. Prior to testing these on the microfluidic device, the ability of these antibodies to identify BRAF mutational status was investigated on culture plates. Supplementary Fig. S6 shows fluorescence images of stained LM-MEL-6 and -53 cells while they were growing on culture plate. Images depicted in Fig. 4a–c show intense red fluorescence cytoplasmic staining with co-localizing DAPI stained nucleus from captured mutant cells (i.e., LM-MEL-6) on *nanoshearing* device. This staining pattern is in agreement with previous reports on cell immunostaining using the same set of antibodies on immunohistochemical samples^41^. In contrast, BRAF wild type melanoma cells (LM-MEL-53 cells) only exhibited typical DAPI staining (Fig. 4d–f). This data demonstrates that *BRAF* V600E mutations could be clearly identified *on-chip* using specific antibodies against the *BRAF* mutant variant. Supplementary Fig. S6 showed staining of LM-MEL-6 and -53 cells while they were growing on culture plate.

## Discussion

Melanoma is a frequently fatal form of skin cancer with the highest incidence in Australia^42^. Considering the recent success rates of targeted and immune therapies in advanced melanoma, it is imperative that highly efficient technologies be employed in the clinic for monitoring treatment response and disease recurrence. An ideal means will be the capture and analysis of CMCs from the bloodstream. Microfluidic based immunoassay platforms have been viewed upon as a promising approach since the ability to induce fluid micromixing (*i.e.* transport of analyte) within the capture/detection domain thereby enhancing the specificity and sensitivity of detection^43^. These have led to the development of robust and efficient detection systems for CTC capture from patients with breast, prostate and colorectal cancer^10^.

In contrast, only limited approaches have been available for the specific isolation of melanoma cells from complex biological samples^23^,^44^. Hou *et al*., developed a polylactic-*co*-glycolic acid-nanofiber embedded nanovelcro chip (PN-nanovelcro chip) capable of specifically isolating melanoma cells expressing CD146 (melanoma cell adhesion molecule, MCAM)^44^. Similarly, Luo *et al*., demonstrated the isolation of melanoma cells from blood samples using a herringbone device coated with MCAM and MCSP antibodies^23^. These approaches rely on hydrodynamic fluid flow to induce fluid mixing with the use of differential geometric arrangements and patterns. In contrast, the use of AC-EHD-induced fluid flow requires minimal operational requirements and simple geometric arrangements where electrode pairs act as both fluid pumps as well the capture domain. The tunable nature of these forces facilitates the manipulation of fluid flow and concomitant fluid mixing (via adjusting AC field) to enhance the number of cell-antibody collisions whilst shearing away nonspecific molecules. With only limited approaches available for the specific isolation of melanoma cells, the device performance achieved in this study is comparable^23^ and in several cases outperforms existing technologies for MCSP based melanoma cell capture from simulated blood or spiked samples using MCSP antibody^20^,^45^,^46^,^47^. Further, our device performance is also comparable with numerous other innovative microfluidic approaches using laminar flow based fluid micromixing to enhance capture efficiency^48^,^49^,^50^,^51^,^52^. However, most of these demonstrations involve EpCAM based CTC capture from different cancer types (e.g., breast, lung, colon, prostrate etc.)

Given the heterogeneity in biomarker expression of melanoma cells during different stages of the disease, the choice of capturing antibody is pivotal in the development of an efficient melanoma cell isolation platform. Ideally, this marker needs to be exclusively expressed by melanoma cells but not by other non-target cells. To date, most common methods for melanoma cell capture, including FDA-approved Cell-Search^TM^, rely on immunocapture using antibodies targeting EpCAM or MCAM markers^10^,^14^,^17^. However, EpCAM is not highly expressed on tumours of mesodermal or neural crest origin, such as melanomas^53^. Moreover, differential expression of melanoma epithelial cell adhesion molecule (MCAM) by other non-melanoma cells such as vascular endothelial cells, smooth muscle cells and pericytes makes it less reliable for melanoma cell isolation^54^. Thus, we selected antibodies targeting MCSP, which is not found on the surface of cells in healthy peripheral blood, while being expressed at least in 85% of melanomas, regardless of the stage^20^,^21^,^22^,^23^,^24^.

Our data also shows that at optimal AC-EHD field conditions (*f* = 600 Hz and *V*_pp_ = 100 mV) MCSP(+) cells could be captured with high efficiency. The optimal AC-EHD condition for melanoma cell capture is different to the optimal AC-EHD condition previously reported for maximum capture of breast cancer cell lines. Thus our device is capable of analyzing different cell types without compromising the capture efficiency by simply adjusting the AC-EHD field.

The presence of non-target cells within the biological media did not compromise efficient detection of melanoma cells. The device’s performance in capturing low number of cells (86% capture for 50–500 cellsmL^−1^) and the purity obtained from PBMCs spiked samples is comparable with existing CTC microfluidic technologies^39^,^52^,^55^,^56^. This indicates that our method could be used for the recovery of low cell numbers from heterogeneous samples containing a large excess of non-target cells. Under optimal conditions, the average recovery for the seed level of 50–500 cellsmL^−1^ was consistent indicating that our method can be used for the efficient recovery of low cell numbers. However, the decrease in capture efficiency for a seed level of 25 cellsmL^−1^ reflects the need for improved shear force manipulation to enhance sensor-target collisions from samples containing limited target entities. We believe further optimization to the experimental protocol and device geometry (length, width, and height of the channel; shape, size, and spacing between electrodes in the long array of asymmetric pairs within the channel) could help to improve capture performance of our device at lower seed levels. Further, these results also align with those obtained using CellSearch^TM^ (*e.g.*, a standard FDA approved method) from spiked melanoma cells^14^. However, in contrast to the high instrumentation and analysis cost involved, our approach involves the use of a simple AC signal generator and efficient microchip.

Finally, our CMC detection approach could easily be coupled with *on-chip* phenotyping of cells and we used the example of testing for the gene-product of mutated *BRAF*. *BRAF* mutations are found in 50–70% of metastatic melanoma patients. Around 80% of those display a valine-to-glutamic acid substitution (V600E) and ~16% harbor a valine-to-lysine substitution (V600K) causing constitutive kinase activation, which can be targeted with existing drug therapies^57^,^58^. Hofman *et al*. first demonstrated the ability to stain for the *BRAF*^*V600E*^driver mutation on patients’ CMCs captured by the ISET system (i.e., filtration- and isolation-by-size technique) using a specific anti-*BRAF*^V600E^ antibody. They demonstrated 100% sensitivity and 81% specificity when results from CMC’s immunohistochemical staining where compared to those from patients’ tumour tissue sequencing^41^. In our study, we demonstrate for the first time, that this approach can also be performed *on-chip* for the evaluation of captured melanoma cells with high specificity. The evaluation of *BRAF*^V600E^ mutation serves as a proof of concept as any immunochemical analysis can be performed in its place. The simplicity of the approach makes it particularly attractive since it could enable assessment of emerging changes without the need for repeated tissue biopsies.

In conclusion, we have developed a simple method for the specific isolation and subsequent enumeration of melanoma cells from complex biological samples. The simplicity and versatility of our approach lies in the (*i*) use of AC-EHD-induced surface shear forces to enhance fluid transport across the capture domain functionalised with a highly effective antibody (i.e. MCSP) whilst removing weakly bound cells or molecules from the electrode surface, and (*ii*) allowing direct immunohistochemical analysis of mutation *on-chip*, thereby representing a simple tool to study melanoma cells post capture. We demonstrated the ability of this approach to detect low cell numbers (average 86% for 50–500 cellsmL^−1^) with improved purity (23.2 ± 1.23%) among large excess of spiked non-target PBMCs or breast cancer cells. We believe this approach could find its relevance as a simple platform for cancer cell isolation of potentially any cancer type. In contrast to other microfluidic-based detection methods that require complicated nanostructures to increase cell capture, our approach allows melanoma cells to be captured using simple arrangement of electrodes pairs and minimal equipment (just the chip and a signal generator). Furthermore, on-chip analysis of *BRAF* mutations can also enable screening of melanoma cells within hours of sampling and circumvent the need for DNA isolation and sequencing, thereby suggesting its potential utility in clinical settings.

## Methods

### Device design and fabrication

In this study, we adopted a simple microfluidic device containing 256 asymmetric planar microelectrode pairs within a long microchannel (Supplementary Fig. S1)^39^. Within each asymmetric electrode pair, the narrow electrode of 100 μm and wide electrode of 400 μm are separated by a distance of 50 μm. The microchannel contains 16 segments connecting each other with 16 electrode pairs in each segment. The characteristic features of this device are: *r*_0_/*d*_2_ = 0.125, *r*_1_/d_2_ = 0.5, *d*_1_/*d*_2_ = 0.25, where *d*_2_ and *d*_1_ are the lengths of electrodes in the pair, *r*_0_ is the distance between the electrodes in the pair, and *r*_1_ is the distance between adjacent electrode pairs. The characteristic feature of this design is to maintain a critical gap between the narrow and wide electrodes in the pair. The distance between the two adjacent electrode pairs, the length of each electrode, and channel dimensions (width (*w*), height (*h*) and length (*l*)) are other key parameters in this design^32^. These electrode pairs present within long serpentine microchannel (*w* = 400 μm; *l* = 196 mm; *h* = 300 μm) containing 16 segments connecting each other, with each segment comprehending 16 electrode pairs. Adjacent electrode pairs in each segment were separated by a distance of 200 μm.

Devices were fabricated at the Queensland node of Australian National Fabrication Facility (Q-ANFF node) as described previously^39^. Briefly, fabrication involved a two-step photolithographic process. Initially, a thin film of negative photoresist (AZnLOF 2070, Microchem, Newton, CA) was spin coated (3000 rpm for 30 s) onto an insulated (silicon oxide layer) silicon wafer (thickness, 1 mm; double side polished) and soft baked briefly at 110 °C for 6 min. Subsequent UV exposure (280 mJ/cm^2^) using a mask aligner (EVG 620, EV Group GmbH, Austria) and development (AZ 726 developer for 3 min) revealed the patterned electrodes. Metallic layers of Ti (20 nm) and Au (200 nm) were deposited using an electron beam (e-beam) evaporator (Temescal FC-2000) under high vacuum conditions followed by acetone lift-off. In the second step, a serpentine channel (*w* = 400 μm, *h* = 300 μm) was constructed on the same wafer containing patterned electrodes by spin coating (1800 rpm) a layer of negative photoresist (Microchem, SU-8 2150). Soft and hard baking steps were performed as per manufacturer’s instructions. Briefly the wafer was soft baked through a series of step change in temperature (65 °C for 7 min → 95 °C for 60 min → 65 °C for 5min). Subsequent UV exposure (380 mJ/cm^2^) was followed by a post-bake step (from 65 °C for 5 min → 95 °C for 20 min → 65 °C for 3 min) and development in propylene glycol methyl ether acetate (PGMEA) for 45 min revealed the fluidic channel. The wafers were then hard baked and diced (ADT 7100 wafer precision dicer) to get individual devices.

### Device functionalization

Capture domain of the device containing an array of gold microelectrode pairs were modified with anti-MCSP using avidin-biotin chemistry (Supplementary Fig. S2). Prior to functionalization, the electrodes were cleaned by sonication in acetone for 5 min, rinsed with isopropyl alcohol and water for another 2 min, and dried with the flow of nitrogen. They were then incubated in biotinylated BSA (200 μgmL^−1^ in PBS, Thermo-Fisher scientific, USA) solution for 2 h followed by coupling with streptavidin (100 μgmL^−1^ in PBS, Invitrogen) for 1 h at room temperature. Subsequently, the electrodes were coated with biotinylated anti-MCSP (5 μgmL^−1^ in PBS, Invitrogen) for another 2 h. These modification steps were performed manually by filling the microchannel with corresponding solution and after each modification step, channel was flushed with PBS (10 mM, pH 7.0) to remove any unbonded molecules.

### Cell culture and labelling

Melanoma cell lines LM-MEL-6, LM-MEL-53, MCSP(+) and LM-MEL-75, MCSP(−) from the Ludwig Institute for Cancer Research (Melbourne, Australia), which have been well characterized and are in early passages have been used^27^. All cell lines were STR-profiled and Mycoplasma tested. Cell lines were maintained in RPMI (Invitrogen) supplemented with 2 mM Glutamax (Invitrogen), 100 UmL^−1^ Penicillin (Invitrogen), 100μgmL^−1^ Streptomycin (Invitrogen), and 10% foetal calf serum (CSL). Breast cancer cells (SK-BR-3) were maintained in DMEM (Gibco) supplemented with 10% foetal calf serum (CSL), 100 UmL^−1^ Penicillin (Invitrogen), 100 μgmL^−1^ insulin and 100 μgmL^−1^ sodium pyruvate. Cells were incubated in 5% CO_2_ at 37 °C. The cultured cells were trypsinised and counted by using a haemocytometer to obtain the desired cell density upon dilution. Cells (100,000 cells/sample) were labelled with 5 μL of DiI+ fluorescent dye (Invitrogen, UK) and incubated at 37 °C for 10 min, followed by PBS wash steps. Fresh donor blood cells were obtained from Australian Red Cross Blood Service and PBMC are extracted using Ficoll-Paque Plus (GE) according to company’s protocol. Cell numbers were counted using haemocytometer. Subsequently, designated concentration of DiI+ labelled cells were then spiked into PBS or PBS containing PBMCs isolated from blood. 500μL of the sample was then placed in to the inlet reservoirs of the devices and driven through the channel by applying AC-EHD field. AC-EHD force was applied for 30 min with 15 min intervals (without AC-EHD) for a total pumping time of 2 h. Captured cells were visualized and counted using fluorescence microscope (Nikon Ti-U upright microscope, Melville, NY). Subsequently cells were fixed by filling the device with cold methanol for 10 min and stained with 4’,6-diamidino-2-phenylindole (DAPI) solution for 5 min. Images were captured using a multichannel fluorescence microscope (Nikon Ti-U upright microscope, Melville, NY) using dual stains (DiI-red and DAPI-blue) and analysed by image processing software (Nikon Ni-S elements, Basic Research). For experiments containing PBMCs, the fluid in the output reservoir was collected and counted using a haemocytometer to calculate the amount of PBMC loss during the experiment.

### Flow cytometry

Flow cytometry was performed on BD Accuri™ C6. Cells were incubated with either anti-MCSP mouse monoclonal antibodies (MAB2585 R&D systems) or isotype-matched control immunoglobulin (Normal mouse IgG sc-2025, Santa Cruz Biotech) prior to staining with labelled secondary antibodies (Alexa Fluor 488 goat anti-mouse IgG antibody, A-11001, Life Technologies). Data were analysed with BD Accuri™ C6 software.

### Anti-BRAF V600E antibody staining

After capturing the melanoma cells on the device, cells were fixed with the IC Fixation Buffer (eBioscience) for 20 min at room temperature. Without washing, cells were permeabilised with the 1× Permeabilisation Buffer (eBioscience) for 5 min at room temperature. The anti-*BRAF* V600E antibody (clone VE1, mouse anti-human IgG2a, Spring Bioscience, USA) was diluted with 1× Permeabilization Buffer (1:50) and 400μL was applied to cover all the channels and incubated at 4 °C for 60 min. The chip is then washed twice with 1× Permeabilisation Buffer. Alexa Fluor 555 conjugated secondary anti-mouse IgG2a antibody (1:1000, Life technologies, USA) was added and incubated at room temperature in the dark for 30 min before analysis using a fluorescence microscope.

## Additional Information

**How to cite this article**: Chang-Hao Tsao, S. *et al*. Capture and *On-chip* analysis of Melanoma Cells Using Tunable Surface Shear forces. *Sci. Rep.* **6**, 19709; doi: 10.1038/srep19709 (2016).

## Supplementary Material

Supplementary Information
