# Supplementary material for: Capture and On-chip analysis of Melanoma Cells Using Tunable Surface Shear forces
Source: Sci Rep. 2016 Jan 27;6:19709. doi: 10.1038/srep19709 (PMC4728558; doi:10.1038/srep19709)
Supplement: Supplementary Information [file srep19709-s1.doc]

**Supporting Information**

Correspondence and request for materials should be addressed to m.shiddiky@uq.edu.au (MJAS), andreas.behren@onjcri.org.au (A.B.) and m.trau@uq.edu.au (MT)

**Capture and *On-chip* analysis of Melanoma Cells Using Tunable Surface Shear forces**

*Simon Chang-Hao Tsao,^2,3,5^ Ramanathan Vaidyanathan,^1^ Shuvashis Dey,^1^ Laura G. Carrascosa,^1^ Christopher Christophi,^3^ Jonathan Cebon ^2,4,5^, Muhammad J. A. Shiddiky^1, §,*^, Andreas Behren ^2,4,5*^, and Matt Trau^1*^.*

1. *Australian Institute for Bioengineering and Nanotechnology (AIBN). University of Queensland, Queensland, 4072, Australia.*
2. *Olivia Newton-John Cancer Research Institute. Heidelberg, Victoria, 3084, Australia.*
3. *Department of Surgery – Austin Health. University of Melbourne, Heidelberg, Victoria, 3084, Australia.*
4. *School of Cancer Medicine- La Trobe University. Melbourne, Victoria, 3086, Australia.*
5. *Ludwig Institute for Cancer Research – Austin Health. Heidelberg, Victoria, 3084, Australia.*

*denotes corresponding authors

*^§^* Present address: School of Natural Sciences, Griffith University (Nathan Campus), Nathan, QLD 4111, Australia


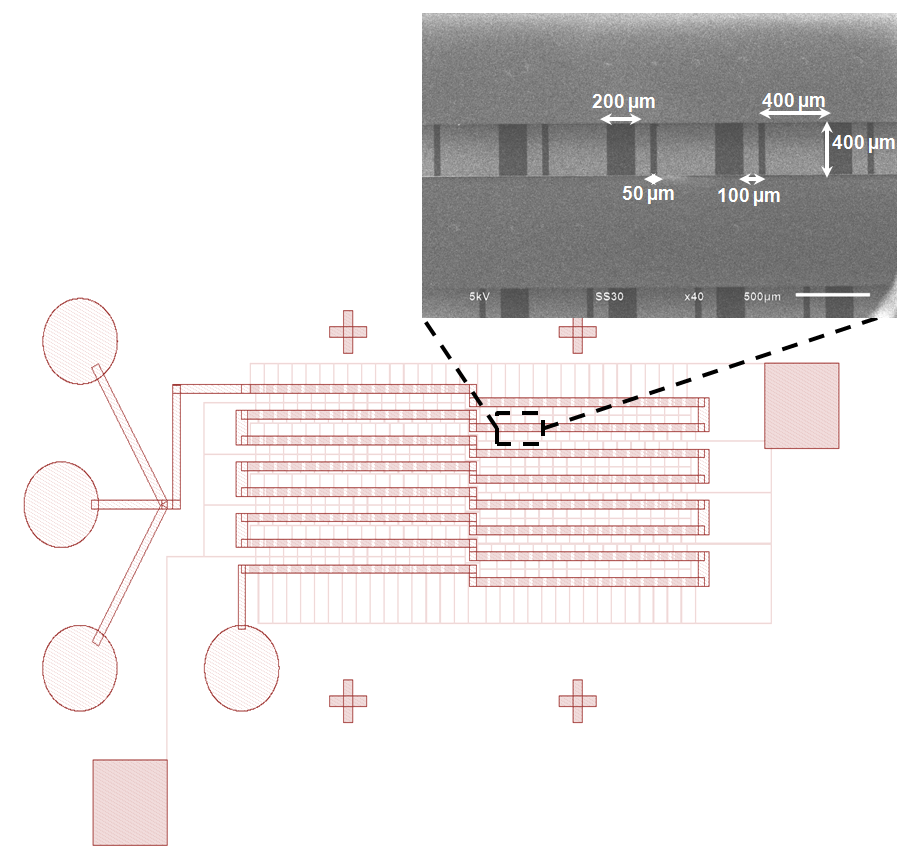


**Supplementary Fig. S1 |Design of melanoma cell capture device.** Schematic representation of microfluidic device containing an array of asymmetric electrode pairs within a microchannel.

**
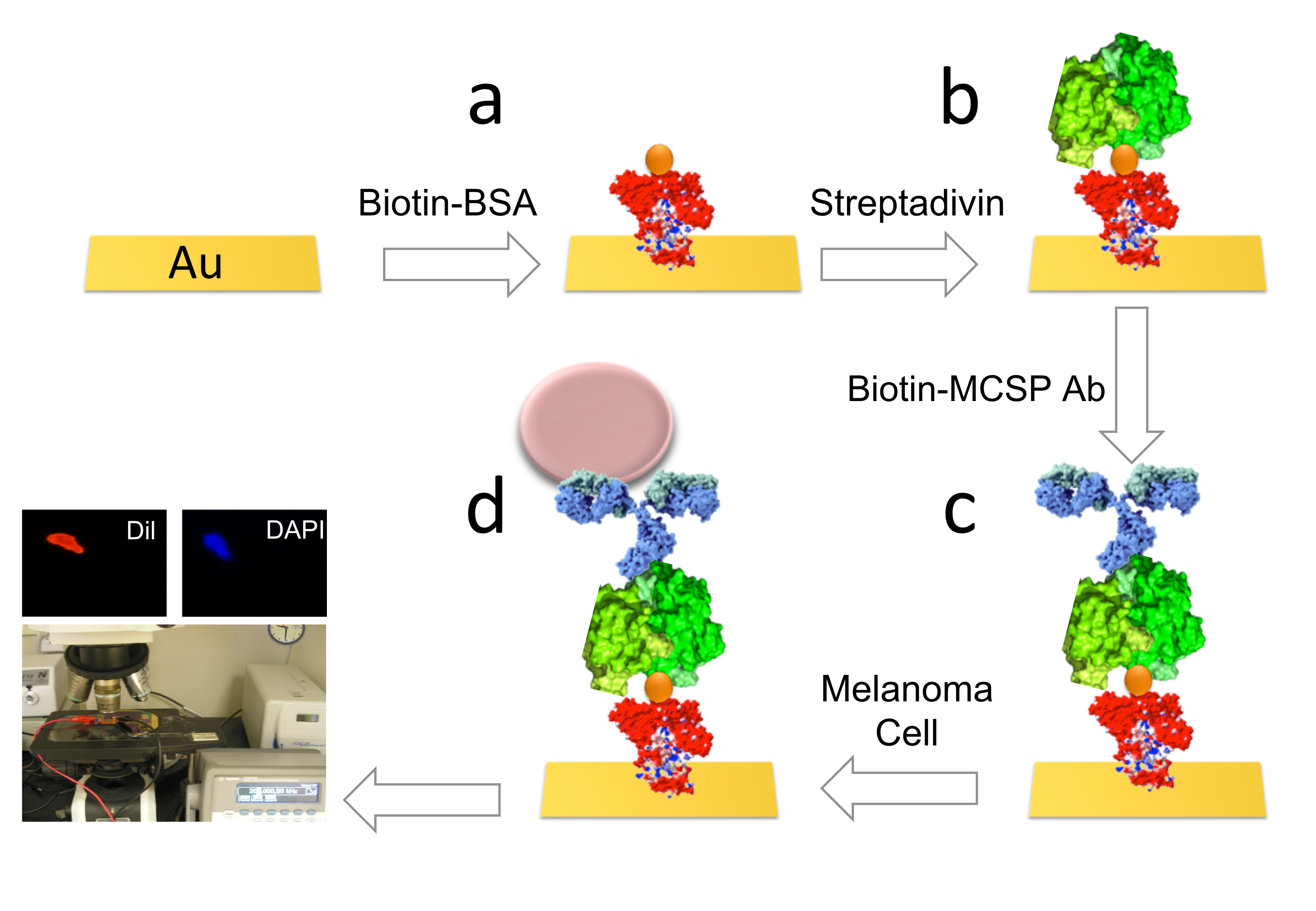
**

**Supplementary Fig. S2 |A schematic drawing of the device functionalization, capture and detection of melanoma cells.** (a) Gold (Au) electrode is covered by biotin-BSA, (b) Binding of streptavidin to biotin-BSA. (c) Biotinylated MCSP antibody binds to the streptavidin. (d) MCSP antibody binds with melanoma cell’s surface MCSP antigen. The MCSP antibody functionalised on the electrode surface captures the melanoma cells by binding to its MCSP antigen. Subsequent immuno-fluorescent staining can be visualized with standard fluorescence microscopy.


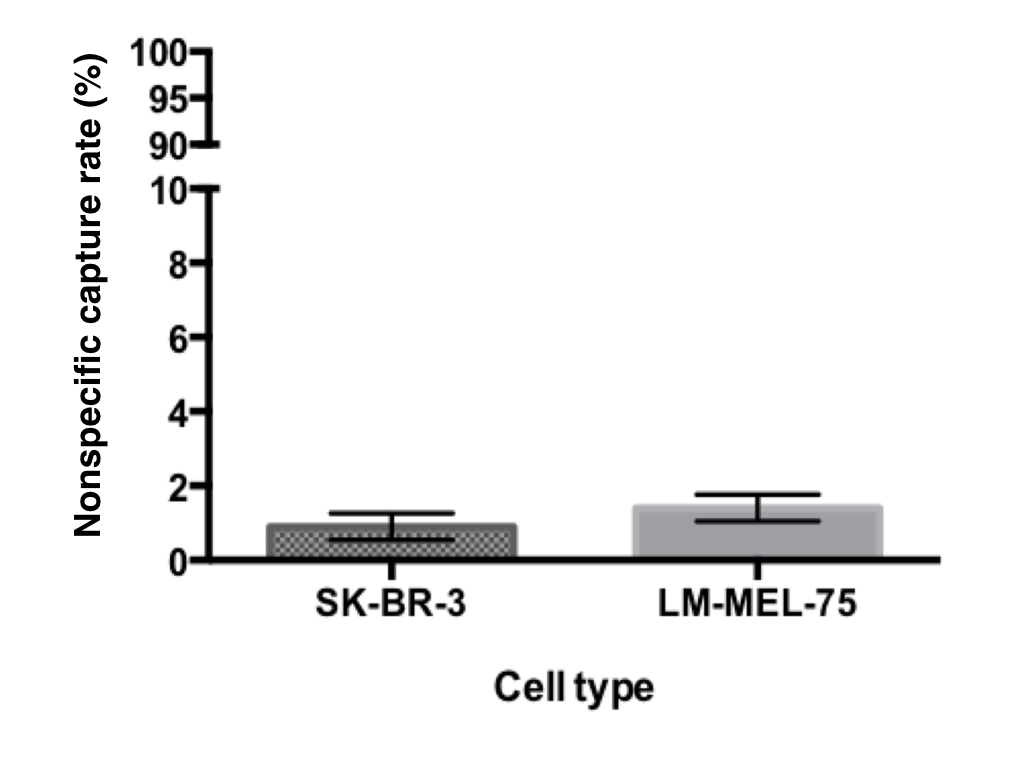


**Supplementary Fig. S3 |Specificity of cell captures**. Capture efficiency from PBS (10 mM, pH 7.4) spiked with pre-stained SK-BR-3 (1000 cellsmL^-1^) and MCSP(-) LM-MEL-75 (1000 cellsmL^-1^) cells in anti-MCSP functionalized device under AC-EHD flow (*f* = 600 Hz and *V*_pp_ = 100 mV). Each data point represents the average of three separate trials *(n = 3)* and error bars represent standard error of measurements within each experiment.


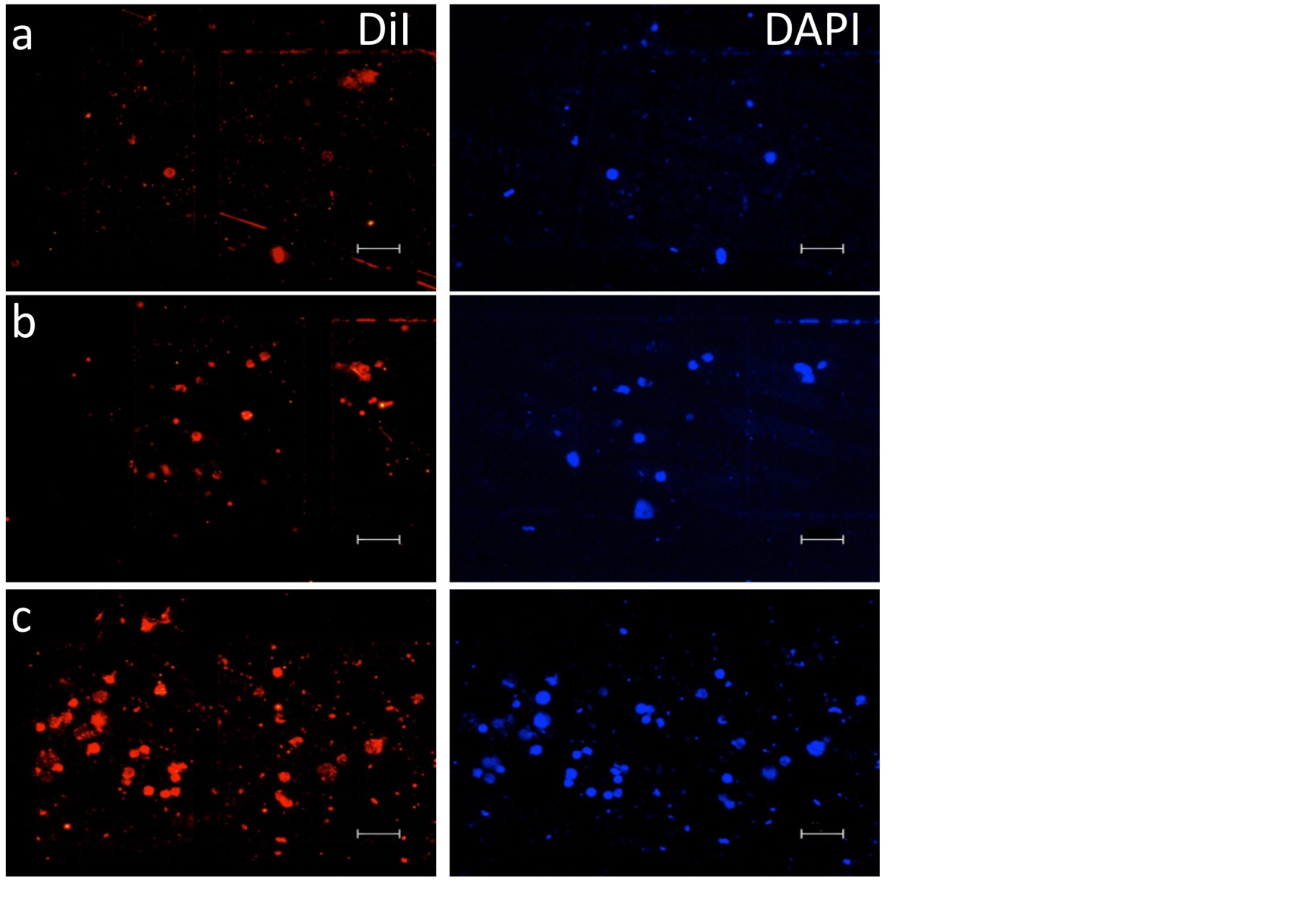


**Supplementary Fig. S4 |Capture performance of AC-EHD device.** Representative images (DiI-cell membrane stain and DAPI-nucleus stain) of different numbers of melanoma cells captured under the AC-EHD field strength of *f* = 600 Hz and *V*_pp_ = 100 mV- (a) 25 cells, (b) 100 cells, (c) 500 cells. 10X magnification. Scale bar is 50 µm.


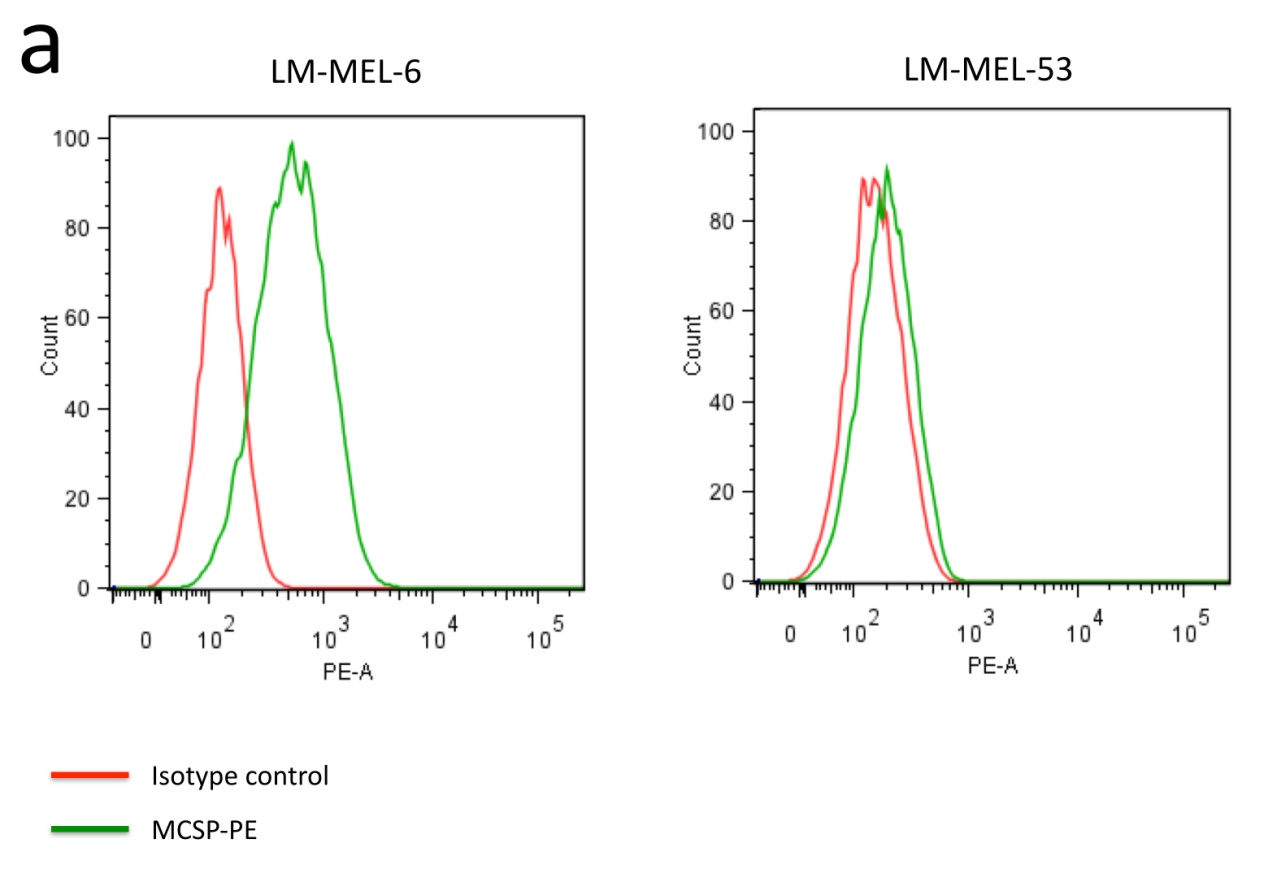


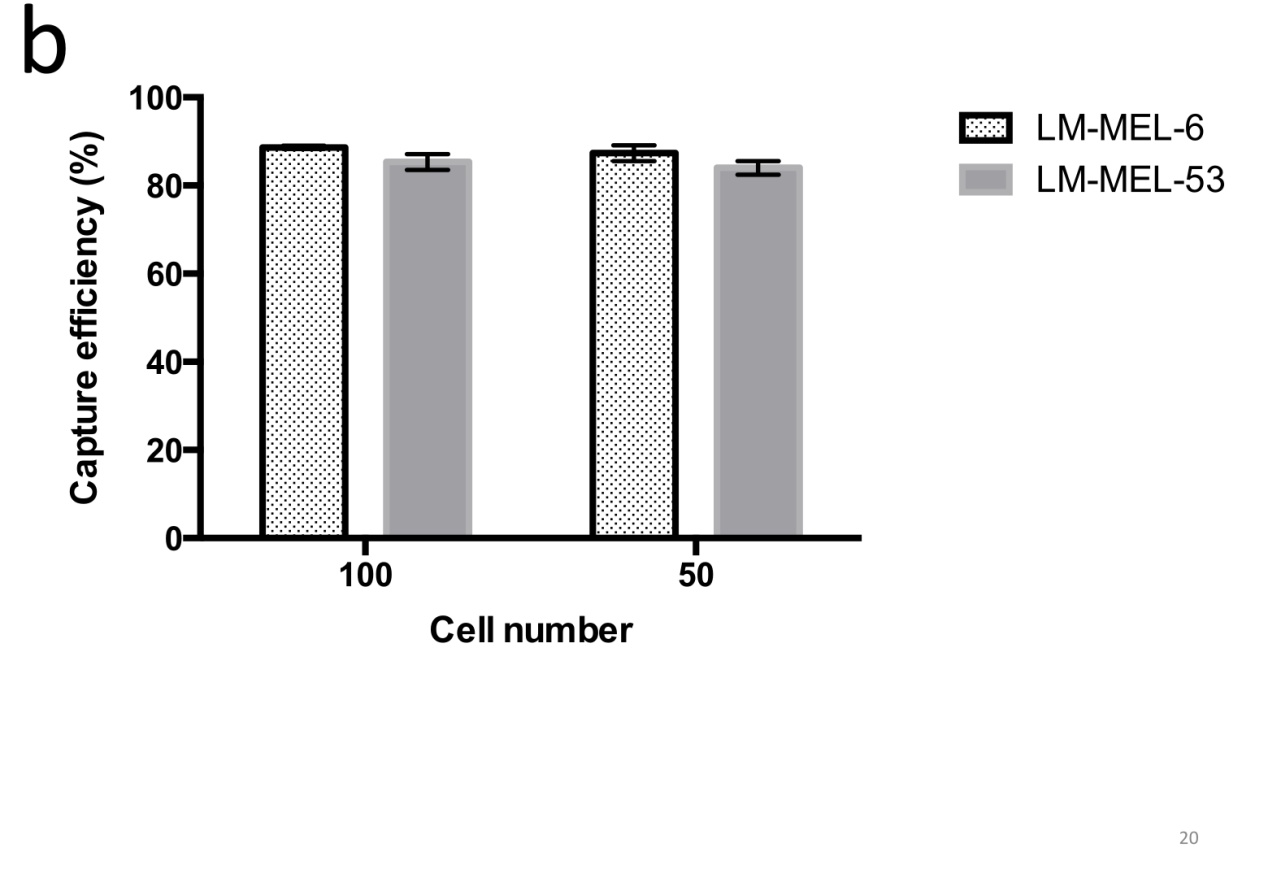


**Supplementary Fig. S5 |Capture efficiency between high and low MCSP expressing cells.**

(a) FACS analysis showing the differences between high (LM-MEL-6) and low (LM-MEL-53) MCSP expressing cells in comparison to isotype control. (b) 100 and 50 of high and low MCSP expressing cells, LM-MEL-6 and LM-MEL-53 respectively, were spiked into PBS and processed through anti-MCSP functionalized device under AC-EHD flow (*f* = 600 Hz and *V*_pp_ = 100 mV).


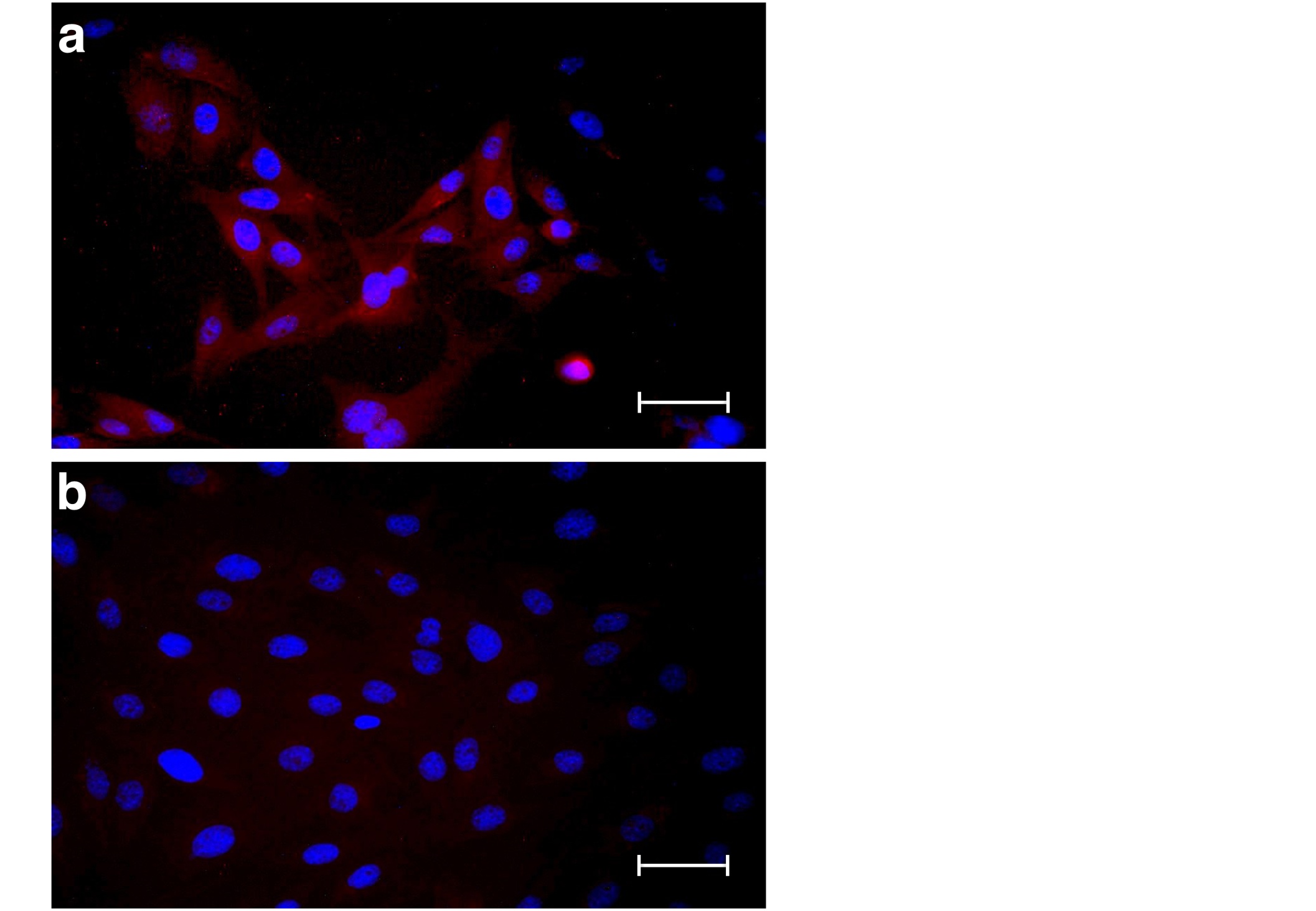


**Supplementary Fig. S6 |Staining of *BRAF^V600E^* on cultured melanoma cells.** (a) *BRAF^V600E^* (+) LM-MEL-6 cells showed strong FITC staining from the anti- *BRAF^V600E^* antibody, whereas (b) *BRAF^V600E^* (-) LM-MEL-53 cells showed no FITC signal and only the nucleus DAPI staining. 20X magnification. Scale bar is 50 µm.
